# 1343. Drug Education Needs of HCPs and Patients During the COVID-19 Pandemic Based on Over 60,000 Unsolicited Medical Information Requests Globally Over 3 Years (2020-2022)

**DOI:** 10.1093/ofid/ofad500.1180

**Published:** 2023-11-27

**Authors:** Sonia K Sandhu, Linda Chen

**Affiliations:** Gilead Sciences, Foster City, California; Gilead Sciences, Foster City, California

## Abstract

**Background:**

The COVID-19 pandemic has resulted in 600 million cases and 6 million deaths globally since December 2019. Medical Information (MI) departments are critical during public health emergencies, providing factual, balanced and non-promotional information to healthcare providers (HCPs) and patients on new treatments, in compliance with industry codes of conduct.

**Figure:**
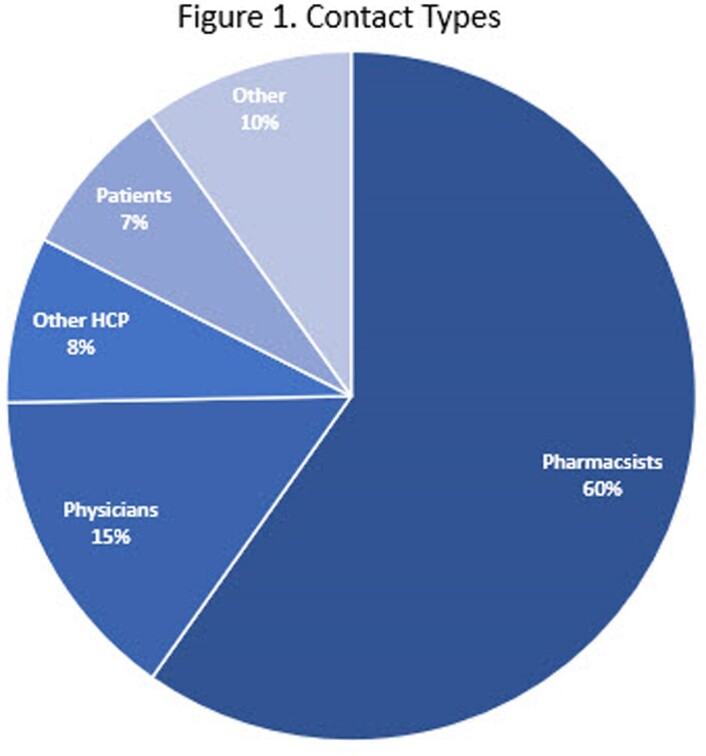

**Methods:**

Unsolicited MI requests for COVID-19 treatment from Jan 2020 to Dec 2022 were analyzed to show the customer journey and evolving needs for drug education. Data gathering and metric analysis were collected from MI databases with interaction dates, countries, contact channels, and responses.

**Results:**

MI received 60,000+ unsolicited requests globally from Jan 2020 to Dec 2022, including 4,400 from patients. Pharmacists (60%) and physicians (15%) were the primary HCPs who contacted MI. Japan and US had the highest number of HCP questions. Changes in HCP questions over time reflected external events such as regulatory approvals and guideline updates, with initial questions related to medication access and clinical trials in 2020, transitioning to medication dosing and preparation in 2021, and new interests emerging in special populations including renal impairment, pediatrics, and pregnancy in 2022. These results suggest that HCPs used MI to help make clinical decisions about which medications are right for their patients.

Patients preferred to contact MI by phone (71%), followed by email (25%) and digital channels (4%). Most patient questions came from US, Mexico, and India. Patients were interested in safety, drug interactions, ingredients, and mechanism of action. Nonclinical topics included drug availability, cost, clinical trial enrollment, and patient assistance programs. Increased patient questions were observed during changes in the medical landscape due to external events (label changes, data updates, and new variants).

**Conclusion:**

Analyzing MI requests is a valuable tool for understanding and meeting the evolving customer needs for drug education. MI insights provide valuable information that informs targeted communications, data-generation priorities to address unmet needs, and educational opportunities that impact clinical decision making and lead to improved patient outcomes.

**Disclosures:**

**All Authors**: No reported disclosures

